# Drug Discovery and Development for Heart Failure Using Multi-Omics Approaches

**DOI:** 10.3390/ijms26062703

**Published:** 2025-03-17

**Authors:** Danielle Rasooly, Alexandre C. Pereira, Jacob Joseph

**Affiliations:** 1 Massachusetts Veterans Epidemiology Research and Information Collaborative (MAVERIC), Veterans Affairs Healthcare System, 150 S. Huntington Ave., Boston, MA 02130, USA; 2 Division of Aging, Brigham and Women’s Hospital, Harvard Medical School, 75 Francis St., Boston, MA 02130, USA; 3 Cardiology Section, VA Providence Healthcare System, 830 Chalkstone Avenue, Providence, RI 02908, USA; 4 Department of Medicine, The Warren Alpert Medical School, Brown University, 222 Richmond St., Providence, RI 02903, USA

**Keywords:** heart failure, drug discovery, genomics, proteomics, transcriptomics, metabolomics, epigenomics, omics, multi-omics, clinical trials

## Abstract

Heart failure (HF) is a complex, heterogeneous syndrome with rising prevalence and high morbidity and mortality. The pathophysiology and diverse etiologies of HF present significant challenges for developing effective therapies. Omics technologies—including genomics, proteomics, transcriptomics, metabolomics, and epigenomics—have reshaped our understanding of HF at the molecular level, uncovering new biomarkers and potential therapeutic targets. Omics also enable insights into individualized treatment responses, the risks of adverse drug effects, and patient stratification for clinical trials. This review explores how multi-omics can enhance heart failure drug discovery and development across all stages of the therapeutic pipeline: (1) target selection and lead identification, (2) preclinical studies, and (3) clinical trials. By integrating omics approaches throughout the drug development process, we can accelerate the discovery of more effective and personalized therapies for heart failure.

## 1. Introduction

Heart failure (HF) affects an estimated 64 million people worldwide and remains a leading cause of morbidity and mortality [1]. Despite advances in HF guideline-directed therapies, HF mortality rates have been increasing since 2012, with a dramatic rise in HF-attributable deaths among young adults [2]. The heterogeneity of HF—stemming from diverse etiologies such as ischemic heart disease, valvular disorders, and cardiomyopathies—coupled with an incomplete understanding of its pathophysiology complicates the development of effective treatments and results in varying responses to therapies among different patient populations.

Although patients with heart failure with reduced ejection fraction (HFrEF) are at higher risk for lower survival and diminished quality of life, these risks can be substantially reduced through the use of guideline-directed medical therapies, including beta-blockers, renin–angiotensin–aldosterone system (RAAS) inhibitors, mineralocorticoid receptor antagonists (MRAs), and sodium–glucose cotransporter 2 (SGLT2) inhibitors—each with a Class 1 recommendation for patients with HFrEF [3]. However, the response to these therapies is not uniform, with certain subpopulations of HFrEF patients experiencing limited to no benefit [4], highlighting the ongoing challenge of identifying effective treatments for all individuals with the condition. This challenge is even more pronounced in HF with preserved ejection fraction (HFpEF), which accounts for nearly half of all HF cases. Currently, no therapies have been proven to reduce mortality in HFpEF [5], and, with the exception of sodium–glucose cotransporter 2 (SGLT2) inhibitors that have demonstrated efficacy for HFpEF and HFrEF, most therapies effective for HFrEF have not demonstrated the same benefits in HFpEF trials [1,6].

Omics technologies, including genomics, proteomics, transcriptomics, metabolomics, and epigenomics, have enhanced our understanding of HF by providing a molecular-level view of the disease. High-throughput omics platforms have generated vast amounts of biological data, enabling insights into the complex interplay of genes, proteins, metabolites, epigenetic modifications, and other omics markers that contribute to the pathophysiology of HF (Table 1). For example, omics-based molecular measurements can be associated with a clinical outcome of interest, such as response to HF therapy, to gain insight into the therapeutic mechanism of action. Multi-omics approaches, which integrate data across multiple omics layers, have the potential to uncover novel biomarkers for early diagnosis, prognosis, and therapeutic monitoring. Importantly, omics can accelerate drug development by identifying novel drug targets and guiding the selection of the most appropriate therapies for individual patients based on their unique molecular profiles.

The use of multi-omics in HF drug discovery and development is particularly beneficial because of the complex, multifactorial nature of the disease. HF is a heterogeneous condition, which makes it challenging to target with a one-size-fits-all approach. Multi-omics enables a more comprehensive and personalized understanding of the disease by identifying molecular drivers of HF. While genomics reveals genetic predispositions associated with HF, these insights, when combined with data from other omics layers—such as proteomics, transcriptomics, metabolomics, and epigenomics—offer a deeper understanding of the disease’s underlying processes. For example, genomics can pinpoint genetic variants associated with HF, but this alone may not explain how these variants manifest at the protein level, how gene expression is altered, or how metabolic processes are affected by the disease. Combining these layers of omics data can allow for the identification of molecular pathways involved in HF, enhancing our understanding of disease progression and uncovering novel drug targets. While this review focuses on the application of multi-omics approaches for HF, the potential of multi-omics extends beyond HF, including cancer [7], neurological disease [8], and infectious disease [9].

**Figure 1:**
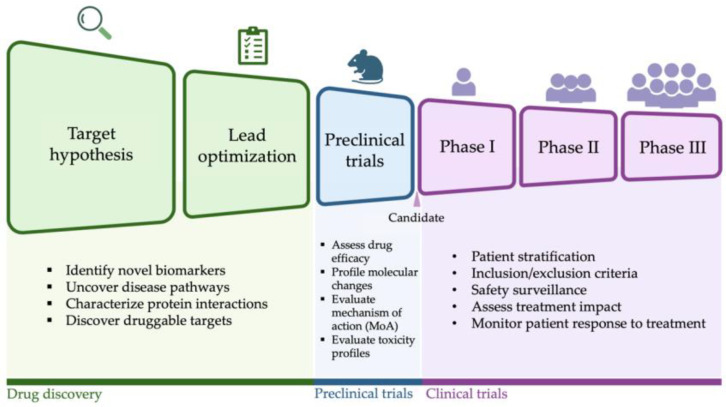
An overview of the various applications of multi-omics in drug discovery and development throughout the therapeutic pipeline.

This review explores how multi-omics approaches can accelerate HF drug discovery and development across all stages of the therapeutic pipeline. Specifically, we examine how multi-omics can enhance (1) target selection and lead identification, (2) preclinical studies, and (3) clinical trials (Figure 1). In the first section, we discuss how multi-omics can facilitate the discovery of novel drug targets by providing insights into the molecular pathways driving HF and prioritizing the most promising candidates for therapeutic intervention. The second section emphasizes the role of omics in evaluating mechanisms of action and predicting adverse effects. In the third section, we examine how multi-omics can enhance clinical trials by identifying biomarkers for patient stratification, enabling more precise clinical decision-making, and optimizing trial design to yield faster, more reliable outcomes. Finally, we highlight key future directions for the field. Ultimately, this review aims to underscore the transformative potential of multi-omics in reshaping the landscape of heart failure therapeutics and accelerating the discovery and development of more effective treatments.

## 2. Target Discovery and Lead Identification

Efficient screening of potential therapeutic targets to select the optimal lead candidate is crucial for advancing drug discovery. The drug development process is costly and high risk, often taking 10–15 years with an average cost exceeding $2 billion for each new drug approved for clinical use [10]. The probability of a drug candidate advancing from Phase I clinical trials to market approval is only 10%, with even lower success rates when considering candidates that make it through preclinical testing [11]. The success of clinical drug development largely depends on selecting the most promising candidates while balancing clinical efficacy, toxicity, and target validation [11]. Rigorous target validation using omics technologies in cell lines and tissues can significantly improve the drug development pipeline, ultimately enhancing the likelihood of success. Genomics, particularly genome-wide association studies (GWAS), play a pivotal role in target discovery by linking genetic variations to disease. GWAS have identified several reproducible genetic loci associated with HF, including *ABO*, *PSRC1*, *LPA*, and *BAG3* [12], which, when further evaluated with transcriptomic and proteomics analysis, can identify promising avenues for target discovery.

### Multi-Omics Approaches for Therapeutic Target Identification

Human genetics-based evidence increases the likelihood of success for drug mechanisms by 2.6 times compared to those lacking human genetics support [13]. In fact, two-thirds (33 out of 50) of the drugs approved by the US Food and Drug Administration (FDA) in 2021 were found to have primary targets supported by human genetics evidence [14]. Complex diseases like HF are partly driven by many common genetic variants, each contributing a modest individual effect on the phenotype. To map the genetic architecture of HF, GWAS [12,15,16] have scanned millions of common variants to identify those with higher frequencies in HF patients compared to individuals without HF. These studies leverage large-scale biobanks that link patient data to genotype information, such as the Veterans Affairs (VA) Million Veteran Program (MVP) [17], one of the largest US-based biobanks, which enhances discovery power in GWAS.

A large-scale GWAS on heart failure meta-analyzed data from 90,653 cases and 1,188,957 control individuals from the MVP and the Heart Failure Molecular Epidemiology for Therapeutic Targets (HERMES) Consortium [12]. The study identified 39 variants with genome-wide significant signals, 18 of which were novel [12]. The association of these significant variants with known HF risk factors and cardiac metrics further strengthens the evidence for their relevance as potential therapeutic targets. Notably, five variants were additionally associated with multiple HF risk factors, including rs9352691/*PHIP*, rs12992672/*TMEM18*, rs4755720/*HSD17B12*, rs233806/*BANK1*, and rs959388/*PRKD1* [12]. Among these, rs4755720/*HSD17B12* was additionally associated with left ventricular end-diastolic volume indexed to body surface area [12]. These findings underscore the potential of GWAS to identify novel genetic variants and molecular pathways that could serve as therapeutic targets.

In MVP studies on HF [12,18,19], HF phenotyping is curated using consensus definitions [20], leveraging International Classification of Diseases (ICD)-9 or 10 codes, and confirming with echocardiograms conducted within 6 months of diagnosis. Additionally, left ventricular ejection fraction values are assessed from various clinical sources, including nuclear medicine reports, cardiac catheterization reports, history and physical examination notes, progress notes, discharge summary notes, and other cardiology notes [12,18,19]. A comprehensive definition of HF ensures robust phenotyping, enhances the reproducibility of findings, and supports the identification of more reliable therapeutic targets. Recent GWAS studies in HF [12,15,16] have primarily used HF incidence as the defining phenotype, which is useful for identifying potential targets for primary prevention. However, exploring alternative phenotype definitions could offer deeper insights into the complexity of the condition. Expanding these analyses to include subsequent events would enhance our understanding of HF progression, helping to identify new targets and prioritize therapeutic interventions for the progression of HF.

Another MVP study investigated the genetic architecture of the two main HF subtypes—HFrEF and HFpEF—to better understand their distinct pathobiology [18]. Using a study population of 19,589 cases with HFpEF, 19,495 cases with HFrEF, and 258,943 control individuals from the MVP cohort, the study identified 13 GWAS loci associated with HFrEF and one locus (*FTO*) associated with HFpEF [18]. Several of the loci were additionally associated with HF risk factors, including *PHACTR1*, *LPA*, and *CDKN2B-AS* with coronary artery disease; *FTO* with body mass index, type 2 diabetes, and high-density lipoprotein (HDL) cholesterol; and *BAG3* with diastolic blood pressure, further supporting their role in HF [18].

Post-GWAS, functional validation using statistical approaches such as Mendelian randomization [21,22] and colocalization [23,24] can help to identify the likely causal variant and the actual causal gene associated with an outcome. This supports the idea that the identified GWAS loci are not only associated with disease risk but also serve as actionable therapeutic targets. For example, using protein quantitative trait loci (pQTLs) from a genome–proteome-wide association study measuring the association between genetic variants and plasma abundances of 4775 distinct protein targets, a study identified 10 putatively causal proteins in HF [12]. Of these, four druggable genes (*MAPK3*, *PRKD1*, *CAMK2D*, and *PRKD3*) encode proteins with serine/threonine kinase activity. Preclinical data validated one of these findings; a calcium/calmodulin-dependent protein kinase II (*CAMK2*) inhibitor administered in transgenic mice carrying a mutation responsible for dilated cardiomyopathy significantly improved cardiac function [25]. This oral inhibitor provides an opportunity to evaluate the causal role of *CAMK2* in clinical trials aimed at preventing HF. Additional druggable genes identified were *APOC3*, *TNFSF12*, and *NAE1*. AKCEA-APO-CIII-L_RX_ is a ligand-conjugated antisense drug comprising an antisense oligonucleotide to apolipoprotein C-III (*APOC3* mRNA) that is liver-specific and may be suitable for long-term use [26].

A subsequent analysis using expression quantitative trait loci (eQTLs) and pQTLs identified causal genes for both HFrEF and HFpEF using Mendelian randomization [19]. Among the druggable genes identified from this analysis amenable for repurposing strategies, *IL6R*, *ADM*, and *EDNRA* emerged as potential targets for HFrEF, and *LPA* was identified as a potential target for both subtypes [19]. The findings, which include well-known HF genes involved in cardiac function as well as newly discovered HF variants influencing inflammation, fibrosis, and metabolic dysregulation, highlight the importance of human genetic studies in identifying novel therapeutic targets [19,27]. For example, *LPA* is a target of several liver-targeted RNA-based therapies in development for cardiovascular disorders [28], and this study replicated the association between *LPA* and HFrEF and demonstrated a suggestive association with HFpEF [19]. The study also rediscovered genes encoding targets for HF-approved drugs, including *ADRB1* for beta-blockers, *SCNN1A* for diuretics, *NR3C2* for mineralocorticoid receptor antagonists, *CACNA1D* for calcium channel blockers, and *SLC5A2* for SGLT2 inhibitors [19]. The study provides comprehensive therapeutic target profiles for initiating new validation programs in preclinical models.

Proteins are the primary targets of almost all drugs, and cataloguing their activities and function is of great value for drug discovery efforts [29]. Further, many proteins perform context-dependent, pleiotropic functions [30], which makes comprehensive evaluation of their forms and functions crucial for designing targeted therapies. Mass spectrometry-based proteomics have advanced from simply cataloguing proteins to enabling in-depth analysis of protein properties, functional modalities, protein interactions, signaling dynamics, and post-translational modifications on a proteome-wide scale [31]. For instance, network analysis was used to analyze the mass spectrometry-based plasma proteomics data of 50 patients with heart failure from the BIOSTAT-CHF (Systems BIOlogy Study to TAilored Treatment in Chronic Heart Failure) Study, revealing that glutathione, arginine and proline, and pyruvate pathways were activated in patients with HF who died or were re-hospitalized, suggesting these as potential therapeutic targets [32]. BIOSTAT-CHF is a large multicenter initiative that has gathered extensive omics data—including genomics, proteomics, transcriptomics, and biomarkers—with the goal of deepening our understanding of HF’s pathophysiology and facilitating the development of targeted therapeutic strategies [33]. The study also highlights the role of glutathione, which has been shown to be depleted by 54% in the left ventricle of failing human hearts. Furthermore, treatment with the glutathione precursor N-acetylcysteine normalized these levels in the left ventricle of rats with chronic HF, improving left ventricular contractile function [34]. This finding underscores the importance of proteomic markers in understanding disease mechanisms and identifying new treatment modalities.

Deriving biological data using mass spectrometry-based platforms, however, is limited by the number of samples that can be processed [35]. While mass spectrometry offers advantages due to its ability to perform shotgun proteomics, aptamer-based proteomic arrays allow for systematic profiling of the plasma proteome and enable inductive discovery without prior hypotheses, thus reducing the risk of type 1 statistical error [36]. However, a challenge of the aptamer-based approaches is their limited ability to detect protein modifications, which are crucial in conditions like HF, where biomarker signaling often depends on post-translational protein modifications.

For example, aptamer-based proteomics has been used to measure the concentrations of 1305 proteins across patient groups at different stages of heart failure [37]. In patients with established heart failure, classical markers such as N-terminal pro-B-type natriuretic peptide (NT-proBNP), C-reactive protein, and troponin T were identified, while 421 proteins were found to be associated with prevalent heart failure [37]. Interestingly, among patients who experienced rapid improvement in cardiac function, 12 of 16 proteins associated with incident HF reverted to levels resembling those of healthy controls, identifying new biomarkers that can be used in the clinical management of patients [37]. In the Framingham Heart Study, aptamer-based proteomic arrays were used to assay 1305 plasma protein levels that were then associated with echocardiographic traits and incident HF [38]. The approach identified three proteins associated with higher HF risk (NT-proBNP, TSP2, MBL) and three proteins associated with lower risk (ErbB1, GDF-8/11, RGMC), highlighting putative biological pathways and protein biomarkers that may be useful for HF risk prevention [38].

Proximity extension assay, a technique that uses pairs of oligonucleotide-labeled antibodies to bind to target antigens, was used to analyze plasma samples in an investigation of three population-based cohorts, revealing several proteomic biomarkers associated with left ventricular hypertrophy, diastolic dysfunction, and incident HF [39]. The study found that NT-proBNP was associated with prevalent left ventricular hypertrophy and diastolic dysfunction. Additionally, increased plasma concentrations of suppression of tumorigenicity-2 (ST2), growth differentiation factor 15 (GDF-15), galectin-4 (Gal-4), and NT-proBNP were associated with incident HF but not consistently with left ventricular hypertrophy or diastolic dysfunction [39]. These findings suggest the involvement of alternative pathways leading to HF and highlight potential therapeutic targets [39]. NT-proBNP and GDF-15 were also identified as having the strongest prognostic value for new-onset HF in a recent study from the UK Biobank, which assessed the association of clinical and proteomic risk profiles for new-onset HF [40]. The study also identified insulin-like growth factor-binding protein 4 (IGFBP4), previously associated with all-cause mortality in patients with acute HF, and WAP four-disulfide core domain protein 2 (WFDC2), which has been strongly associated with HF severity [40].

In addition to genomics and proteomics, transcriptomic profiling has been used to explore the role of gene expression in biological function and identify therapeutics and dysregulated pathways contributing to heart failure susceptibility. For example, transcriptome analysis of 944 patients with chronic heart failure from the BIOSTAT-CHF study identified 1153 genes (6.5%) that were differentially expressed between survivors and non-survivors, with 557 genes up-regulated and 596 down-regulated [41]. Among these findings, an altered T-cell molecular profile was a key feature in non-survivors [41]. For example, T-cell receptor (TCR) signaling molecules CD3 gamma and epsilon, along with genes encoding their protein adaptors and the CD28 co-stimulatory signals, were down-regulated in non-survivors [41]. The study suggests that these mortality-associated transcripts can potentially be reversed in vitro by drugs, with further analysis pointing to fibroblast growth factor 23 (FGF23) and soluble suppression of tumorigenesis-2 (sST2) as promising therapeutic targets [41]. Specifically, antagonizing FGF23, linked to increased left ventricular mass [42] and incident heart failure [43] as well as left ventricular systolic dysfunction [44] and down-regulating sST2 expression, or its interaction with IL-33 receptors could offer new avenues for treatment [41]. Transcriptome signatures offer a promising strategy for identifying potential therapeutics and pathways that modulate disease processes in experimental models. For instance, in a mouse model of diet-induced dyslipidemia and atherosclerosis, drug treatments that restored gene expression patterns to normal levels were associated with favorable physiological outcomes [45], further supporting the role of transcriptomic data for drug repurposing and discovery.

Metabolomic profiling uncovers the underlying pathophysiological processes related to energy homeostasis and metabolism, which are important for HF target discovery due to the heart’s high metabolic demand. In the HF-ACTION (Heart Failure: A Controlled Trial Investigating Outcomes of Exercise Training) clinical trial, which focused on chronic systolic HF patients, metabolomic profiles were associated with adverse outcomes and assessed for changes in response to treatment for end-stage systolic HF [46]. The study identified a metabolite factor predominantly composed of long-chain acylcarnitines, which were independently associated with a lower peak V0_2_ and both primary and secondary clinical endpoints [46]. Notably, these metabolite levels decreased with circulatory support, which suggests that mitochondrial-based therapeutics may offer promising treatment options for systolic HF [46]. These findings highlight the potential of metabolomics approaches in identifying novel treatment strategies for HF.

Integrating multiple layers of omics data provides a comprehensive approach to target discovery and enables a deeper understanding of HF pathophysiology. In a recent study, successive prioritization of omics layers—proteome, phenome, and myocardial transcriptome at single-nucleus resolution—was used to identify potential HF targets. The study, which included over 50,000 individuals from Coronary Artery Risk Development in Young Adults (CARDIA), Framingham Heart Study, and UK Biobank, identified circulating proteomic signatures and echocardiographic pathophenotypes associated with HF [47]. By focusing on structural and functional associations with the proteome, the study prioritized genes historically implicated in hypertrophy (e.g., *IL1RAP*, *IL1R1*, *ADAM23*, *DKK3*) and fibrosis signaling (e.g., *IGFBP4*, *MMP2*, *SERPINE1*, *POSTN*), identifying potential therapeutic strategies that merit further investigation [47].

## 3. Multi-Omics Approaches in Preclinical Studies for Heart Failure

Preclinical drug discovery typically takes 5.5 years and accounts for one-third of the cost of drug development [48]. This stage involves evaluating the mechanism of action (MoA) and the safety of potential therapeutic interventions. For example, transcriptomics can identify changes in gene expression that may signal drug-induced toxicity, such as altered mRNA expression profiles associated with adverse cardiac effects [49]. Metabolomics can detect subtle metabolite changes prior to toxic events, helping to identify early biomarkers of toxicity [49]. Proteomics, particularly through mass spectrometry, can reveal changes in protein expression and post-translational modifications in response to drug exposure [50]. For instance, proteomics can detect alterations in liver-specific proteins, such as those involved in metabolism and detoxification processes, which are key indicators of hepatotoxicity and important in HF drug discovery. These protein biomarkers can identify early signs of liver damage, such as oxidative stress [50]. This section discusses several applications of multi-omics in preclinical HF studies.

### 3.1. Evaluating Drug Mechanism of Action

Multi-omics approaches can reveal how a drug modulates molecular pathways in HF, revealing its on-target effects as well as potential off-target consequences.

As an example of a metabolomics approach, in the DEFINE-HF (Dapagliflozin Effects on Biomarkers, Symptoms and Functional Status in Patients With HF With Reduced Ejection Fraction) trial, which studied the sodium–glucose cotransporter 2 (SGLT2) inhibitor dapagliflozin in HFrEF, mass spectrometry profiling of 63 plasma metabolites at baseline and after 12 weeks showed increased ketone-related and short-chain acylcarnitine and medium-chain acylcarnitine metabolite clusters defined by principal component analysis [51]. These metabolic shifts suggest that SGLT2 inhibitors may induce metabolic reprogramming and enhance mitochondrial function [51]. In non-diabetic rats with left ventricular dysfunction after myocardial infarction, the effect of SGLT2 inhibition with empagliflozin resulted in metabolic changes associated with an increase in cardiac ATP production, including diminished interstitial fibrosis, reduced myocardial oxidative stress, reduced mitochondrial DNA damage, and increased circulating ketone levels [52].

As an example of an epigenetic approach for evaluating drug mechanism of action, one study demonstrated that empagliflozin may prevent hyperglycemia-induced demethylation changes in human ventricular cardiac myoblasts [53]. SGLT2 gene silencing experiments further suggested that blocking SGLT2 might reduce DNA demethylation, pointing to a potential new mechanism of action in which SGLT2 can exert cardio-beneficial and anti-inflammatory effects [53].

### 3.2. Assessing Drug Safety in Preclinical Models

Adverse drug events pose a challenge to treatment efficacy and are a frequent reason for poor drug compliance [54]. The early detection of potential drug-related risks is crucial for improving the safety profile of new therapies. Multi-omics technologies, including whole genome sequencing and transcriptomic profiling, can predict adverse reactions by identifying omics profiles that may predispose individuals to toxicity. For example, a study investigated the use of drug-induced gene expression profiles for uncovering mechanisms of cardiotoxicity in tyrosine kinase inhibitors [55]. The analysis used transcriptomic data from human induced pluripotent stem cell-derived cardiomyocytes to identify drug-specific gene expression patterns for 54 drugs [55]. The study found that cardiotoxic tyrosine kinase inhibitors affect cellular pathways related to energy metabolism and contractility; integrating the mRNA expression data with genomic, pathway, and single-cell transcriptomic datasets resulted in the identification of signatures for drug cardiotoxicity [55]. The study demonstrates that multi-omics can generate reliable pathway signatures and serves as a hypothesis generator for identifying drug-induced adverse events [55].

In another study, patient-specific human induced pluripotent stem cell-derived cardiomyocytes were used to screen 21 FDA-approved tyrosine kinase inhibitors (TKIs) for cardiotoxicity [56]. By integrating measurements of cardiomyocyte viability, contractility, electrophysiology, calcium handling, and signaling, a ‘cardiac safety index’ was used to assess the cardiotoxicity of existing TKIs [56]. The study found that three out of the four TKIs with the lowest safety indices were VEGFR2/PDGFR-inhibiting TKIs, known to cause cardiovascular toxicities such as hypertension, QT prolongation, and heart failure [56]. Similarly, transcriptomic profiling of human primary cardiomyocyte-like cell lines was used to predict the cardiotoxicity of 26 FDA-approved kinase inhibitors [57]. This approach demonstrated how comparing transcriptomic responses with clinical cardiotoxicity risk scores can help identify genes predictive of kinase inhibitor-associated cardiotoxicity, aiding in drug development by ranking the relative risks of novel inhibitors [57]. In another recent study, a multi-omics approach integrating toxicoproteomics and toxicometabolomics data identified key metabolic and biochemical pathways—particularly those related to energy metabolism and in the glutathione system—associated with thiazolidinedione-induced cardiotoxicity [58]. This analysis provides new insights into the pathways involved in drug-induced HF and suggests strategies to improve the safety profile of thiazolidinedione agents [58].

## 4. Multi-Omics Approaches in Clinical Trials for Heart Failure

Clinical trials are the cornerstone of drug development, bridging the gap between preclinical findings and patient-based outcomes. Traditional clinical-trial methodologies often struggle to account for the heterogeneity of heart failure, complicating the identification of effective treatments for diverse patient populations [59]. Clinical trials designed for primary HF prevention focus on identifying early interventions that can reduce the risk of developing HF in at-risk populations, while secondary prevention trials aim to modify disease progression and reduce the risk of adverse outcomes in individuals already diagnosed with HF. These distinct trial goals influence the types of biomarkers and omics technologies that are most relevant—such as early-stage prognostic markers in primary prevention versus disease-modifying biomarkers in secondary prevention. Multi-omics approaches can enhance the design and interpretation of both types of trials by providing more comprehensive insights into the underlying molecular mechanisms and therapeutic targets. These approaches can offer new avenues for predicting patient response to therapy, personalizing treatment plans based on a patient’s unique omics profile, and aiding in biomarker discovery [60]. For example, genomics data can enhance patient stratification in clinical trials by identifying genetic variants that influence disease risk and treatment response, enabling the classification of patients into subgroups that are more likely to benefit from a specific therapeutic strategy. Polygenic risk scores derived from genetic data have shown potential in identifying subsets of HF patients who respond favorably to treatments like beta-blockers, thus improving clinical trial outcomes. This section explores the applications of multi-omics in HF clinical trials, including improving patient selection, monitoring treatment responses, tracking disease progression, and identifying potential surrogate endpoints.

### 4.1. Enhancing Patient Stratification

HF clinical trials face the challenge of patient population heterogeneity, with clinical presentations and outcomes shaped by a wide range of cardiac and non-cardiac comorbidities [61,62]. This complexity challenges efforts to predict treatment responses. Traditional clinical approaches commonly use a “one-size-fits-all” strategy, applying the same therapies to all patients based on limited clinical data [63]. A study evaluating the efficacy of emerging HF therapies (sacubitril/valsartan, dapagliflozin, empagaliflozin, vericiguat, or omecamtiv mercabil) in distinct subgroups of HFrEF patients found that the clinical benefits of each drug are tailored to specific patient groups [64]. For instance, dapagliflozin, empagliflozin, and sacubitril/valsartan were more effective than vericiguat and omecamtiv mercabil in reducing the primary outcome of CVD and HF hospitalization in patients over 65 years old, with dapagliflozin showing the highest efficacy [64]. The study also revealed that SGLT2 inhibitors empagliflozin and dapagliflozin significantly reduced the primary outcome in patients with diabetes, while all three therapies—dapagliflozin, empagliflozin, and omecamtiv mercabil—reduced the primary outcome in patients with ischemic HFrEF, with dapagliflozin demonstrating the largest reduction in primary endpoint [64]. Additionally, dapagliflozin followed by sacubitril/valsartan and vericiguat, compared to the other therapies, showed the largest reduction in primary outcome in patients with HFrEF and chronic kidney disease (eGFR < 60 mL/min), which is particularly noteworthy given that HF therapies may potentially have detrimental effects on renal function [64]. Therefore, tailoring drug therapies to specific patient populations in clinical trials and in real-world settings can optimize treatment outcomes.

The 2019 FDA guidance emphasizes the utility of biomarkers for enrolling heart failure (HF) patients at higher risk of adverse events, stratifying them based on their predicted prognosis, and facilitating early-stage proof-of-concept and dose-selection studies [65]. Multi-omics enables more precise stratification by grouping individuals based on molecular profiles rather than clinical features [66]. This refinement can improve clinical trial success by allowing for more targeted therapies, which may offer better translation into clinical practice, addressing both the heterogeneity of HF and the limitations of current trial designs. A notable example is the GENETIC-AF trial (Genotype-Directed Comparative Effectiveness Trial of Bucindolol and Toprol-XL for the Prevention of Symptomatic Atrial Fibrillation/Atrial Flutter in Patients with Heart Failure), which compared the efficacy of bucindolol and metoprolol succinate in maintaining sinus rhythm in HFrEF patients with the ADRB1 Arg389Arg genotype [67]. GENETIC-AF is an example of a randomized controlled trial that incorporated an adaptive trial design, which used data accumulated during the trial to change the study features. The study demonstrated that genomic phenotyping could identify a proportion of HF patients who exhibit a differential drug response in preventing atrial fibrillation, showcasing how genomic information can be used to guide clinical trial design by identifying patient subgroups who would respond better to a particular drug [67].

Biomarker testing has become a crucial component in the assessment and management of heart failure (HF), with several markers having been explored over time. Among these, natriuretic proteins, including B-type natriuretic protein (BNP) and its precursor N-terminal pro-B-type natriuretic peptide [NT-proBNP], remain the gold standard for clinical HF assessment. BNP and NT-proBNP are extensively used as inclusion criteria in early-phase HF clinical trials [68,69,70,71] to ensure appropriate patient selection and enhance event rates [72]. A clinicaltrials.gov database analysis identified 3446 HF trials, of which 365 (10.6%) used BNP or NT-proBNP as inclusion criteria (and 43% of the 365 used both) [68]. In the PARAGON-HF trial, the 193 patients (4.0%) who did not meet the final natriuretic peptide-based inclusion criteria (NT-proBNP > 300 ng/L for patients in sinus rhythm or >900 ng/L for those in atrial fibrillation/flutter) showed a lower rate of HF hospitalizations and cardiovascular death compared to those who did meet the criteria [73].

Recent updates to clinical guidelines also highlight the growing role of proteins such as troponin, soluble (s)ST2, and galactin-3 in HF pathogenesis [74]. These protein biomarkers enhance patient stratification by predicting disease trajectories and identifying higher-risk patients, which can reduce type II errors in underpowered studies [75,76].

In addition to proteomics, genomics can assist in patient stratification by identifying genetic variations that influence disease risk and treatment response, allowing for the classification of patients into subgroups that are most likely to benefit from a specific therapeutic intervention. For example, a polygenic risk score derived using data from 1436 patients with HFrEF distinguished patients who derived substantial survival benefits from beta-blocker treatment from those who did not [77]. If validated in clinical trials, this genetic profile could help identify the subset of HFrEF patients most likely to benefit from beta-blocker therapy, sparing a portion of patients from unnecessary treatment [77].

Multi-omics can enhance clinical trial design by allowing patient stratification based on not only clinical characteristics but also their molecular profiles. By integrating omics data, clinical trials can pinpoint subgroups that are more likely to respond positively to a therapy based on their unique molecular makeup. This approach improves trial design by refining trial inclusion and exclusion criteria, enabling more targeted enrollment, and reducing heterogeneity that may obscure treatment effects. In practical terms, this could lead to smaller, more efficient trials by focusing on patient populations that are most likely to benefit from the therapeutic.

### 4.2. Monitoring Treatment Response and Disease Progression

Omics can identify molecular signatures associated with therapeutic benefit or disease progression, facilitating earlier and more accurate adjustments to therapeutic interventions. Circulating microRNAs (miRNAs), which are small noncoding RNA molecules that regulate gene expression and play a critical role in cardiac remodeling—including myocyte hypertrophy, excitation–contraction coupling in the cardiomyocyte, increased myocyte loss, and myocardial fibrosis—have gained attention as potential novel biomarkers in HF for monitoring response to therapy [78]. For example, it has been shown that Myh7b/miR-499 expression and miR-423-5p plasma levels were reduced in response to oligonucleotide-based therapies, resulting in improved cardiac function [79]. Such biomarkers may offer an early indication of therapeutic efficacy, helping to guide clinical decisions in real time.

The study also revealed the role of several differentially expressed proteins involved in arginine and proline metabolism, including 4-trimethylaminobutyraldehyde dehydrogenase (ALDH9A1), prolyl 4-hydroxylase subunit alpha-1 (P4HA1) and SRM [34]. In a previous study on patients with HF, L-arginine decreased heart rate, mean systemic arterial pressure, and systemic vascular resistance and increased right atrial pressure, cardiac output, and stroke volume, which suggests that L-arginine improves cardiac performance by acting on systemic vascular resistance [80]. The proteins ALDH9A1, dihydrolipoyl dehydrogenase mitochondrial (DLD), and GLO1 were also identified, all of which play a role in pyruvate metabolism dysregulation [34]. It has previously been demonstrated that delivering intracoronary pyruvate into the left main coronary artery of patients with congestive HF resulted in an increase in cardiac index and stroke volume index and a decrease in pulmonary capillary wedge pressure and heart rate [81].

Another study utilized a multi-omics approach, integrating genetic, transcriptomic, and proteomic data to identify key pathways associated with HF progression leading to mortality [82]. Using machine learning techniques based on gradient boosting and stacked regularization, the study analyzed multi-omics data from 2516 HF patients [82]. The study revealed four key pathways associated with HF progression: PI3K/Akt, MAPK, Ras signaling pathway, and epidermal growth factor receptor tyrosine kinase inhibitor resistance [82]. Notably, these pathways are known to be strongly related to each other. Further, disruption of ERBB family receptors impairs downstream signaling to the Ras–ERK and PI3K/Akt pathways, which suggests that neuregulins, which stimulate ERBB receptors tyrosine kinases, may be a beneficial therapeutic strategy [83].

The platelet proteome is another resource for identifying proteins involved in HF progression, as platelets both contribute to and respond to inflammatory processes [83]. A study comparing platelet proteomes from hospitalized and outpatient HFpEF patients and validated with iPSC-derived cardiomyocytes revealed elevated levels of serum amyloid A (SAA), lipopolysaccharide binding protein, apolipoprotein A1, and S100A8—proteins known to be involved in inflammatory pathways—contributing to HF progression [84].

In another study, higher levels of proteomics-based soluble urokinase plasminogen activator receptor were significantly associated with the risk of incident all-cause, ischemic, and nonischemic HF risk, independent of NT-proBNP levels, CRP levels, and demographic and risk factor characteristics, which suggests that these levels may be valuable prognostic information [85].

Interindividual variability in drug response is influenced by a range of factors, with genetics playing a key role in modifying the activity or availability of drug-metabolizing enzymes, receptors, channels, or other proteins that play a role in pharmacokinetics and pharmacodynamics [86]. Pharmacogenomics can optimize treatment monitoring by refining drug regimens and predicting how patients will metabolize or respond to specific therapies. Interindividual variation has been demonstrated in response to beta-blocker therapy [87], which has been a cornerstone of HF treatment for over 20 years, and was demonstrated to be efficacious in the landmark clinical trials COPERNICUS (Carvedilol) [88], MERIT-HF (Metoprolol) [89], and CIBIS-II (Bisoprolol) [90]. Genetic variation in the adrenergic receptors β1-AR (*ADRB1*), β2-AR (*ADRB2*), and α2C AR (*ADRA2C*) [91] may explain the variability in pharmacologic response to beta-blocker therapy.

### 4.3. Accelerating Drug Development Through Early Endpoint Detection

Omic markers can identify potential surrogate endpoints in HF clinical trials that can be reached more quickly than composite endpoints, enabling shortened follow-up periods and trial duration and, thus, a lower overall cost of trials [92]. However, therapies have a multitude of effects beyond the surrogate endpoint, which can result in neutral phase III trials followed by successful phase II trials with a surrogate endpoint; the complexity of the relationship between intermediate markers and long-term clinical outcomes makes identifying reliable surrogate endpoints challenging [93].

Although there are no universally accepted surrogate endpoints for HF phase II trials, NT-proBNP is commonly used as a marker [94], despite challenges in its reliability and predictive ability [95]. For example, an analysis of 16 phase III chronic HF trials found that therapy-related changes in natriuretic peptides were modestly correlated with HF hospitalizations but not with all-cause mortality [96]. The phase III ASTRONAUT (Aliskiren Trial on Acute Heart Failure Outcomes) trial demonstrated that aliskiren reduced NT-proBNP levels over 12 months of follow-up but had no impact on mortality or hospitalization endpoints [97]. Moreover, the effectiveness of NT-proBNP as a surrogate endpoint may be complicated by patient comorbidities, as demonstrated in the ASTRONAUT trial, where the ability of aliskiren to lower NT-proBNP varied based on the presence of atrial fibrillation and flutter [98].

The need for novel biomarkers beyond NT-proBNP is critical for developing reliable surrogate endpoints and guiding therapeutic strategies. Surrogate endpoints are particularly valuable in early-phase clinical trials, as they reduce sample size requirements, minimizing exposure to potentially ineffective or even harmful interventions. Furthermore, these endpoints provide crucial mechanistic insights into the effects of therapeutic interventions. In the BIOSTAT-CHF cohort, a study of 1040 patients with HFrEF explored the relationships between changes in the plasma concentrations of 30 biomarkers and subsequent morbidity and mortality, assessing their potential as surrogate endpoints in phase II HFrEF trials [99]. Notably, changes in NT-proBNP and the four-disulfide core domain protein HE4 (WAP-4C) were independently associated with reduced hospitalization and mortality risk, with NT-proBNP emerging as the strongest predictor [99]. However, there are limitations to using NT-proBNP, as it may not accurately reflect treatment efficacy. For example, in the PARADIGM-HF trial, which evaluated the effects of the angiotensin receptor–neprilysin inhibitor (ARNI) sacubitril/valsartan in patients with HFrEF, NT-proBNP levels increased in patients receiving the therapy, despite significant reductions in HF hospitalizations and mortality [100]. This discrepancy suggests that NT-proBNP’s role as a reliable indicator of treatment response could be more nuanced, complicating its utility as a surrogate endpoint in HF clinical trials.

Troponin levels are associated with poor prognosis in patients with acute decompensated HF, with elevated levels indicative of disease severity; as a result, troponin has been explored for its prognostic value in trials [101]. In the RELAX-AHF trial for serelaxin, baseline, peak, and peak change in high-sensitivity troponin T were associated with poor patient outcomes [102], and in a post hoc analysis, patients with baseline levels below the 99th percentile were at significantly reduced risk for cardiovascular death through day 180 [103]. Similar results were shown in the PARADIGM-HF trial for chronic HF, where patients receiving the angiotensin–neprilysin inhibitor LCZ696 exhibited consistently reduced levels of NT-proBNP and troponin [100].

The increasing frequency of positive phase II trial results followed by negative phase III trial outcomes suggests that the traditional approach of using surrogate endpoints to predict phase III success in HF trials may need to be refined. A more tailored strategy focusing on drug–patient interactions and mechanistic insights may be key for better predicting phase III treatment responses and improving the design of HF clinical trials [95]. Unbiased omics methodologies can help uncover biomarkers with responses that closely align with the drug’s biological effects being studied [104].

## 5. Current Limitations and Future Perspectives

### 5.1. Translational Challenges in Advancing Omics from Bench to Bedside

Evaluation of the applicability of omics-based biomarkers for clinical practice or use in clinical trials requires careful consideration of the supporting evidence for their analytical and clinical validity, as well as addressing key challenges related to regulatory issues, data privacy, data standardization, and financial concerns. The primary barrier to translating omics-based biomarkers to routine clinical practice is ensuring their rigorous clinical validation. Large multicenter clinical trials are necessary to confirm the clinical utility of these biomarkers across diverse patient populations. Additionally, the development of more affordable omics platforms and faster processing methods will assist in scaling omics-based biomarkers for routine clinical use.

Several challenges exist in incorporating multi-omics into clinical trials, including issues in data standardization, data privacy, regulatory approval, and financial considerations. Variations in sample collection, processing, and analysis can challenge the reliability and reproducibility of omics findings. To address this, the establishment of internationally recognized laboratory standards and standardized operating procedures for data collection and processing can help to ensure the more effective integration of omics biomarkers into clinical trials and clinical practice. Furthermore, health data generated by omics technologies must be securely stored in compliance with relevant privacy laws and regulations to protect patient confidentiality. As omics technologies continue to evolve, regulatory bodies have yet to establish comprehensive guidelines for the approval of omics-based biomarkers. Clear regulatory pathways are necessary to facilitate the integration of omics-based biomarkers into clinical trials.

Finally, financial considerations are significant, including the high costs associated with generating omics data, performing analysis, and maintaining the necessary infrastructure for data security and handling. Addressing technological, regulatory, financial, and privacy barriers will be key in unlocking the full potential of omics-based biomarkers in both clinical practice and clinical trials.

### 5.2. Future Perspectives

As the field of multi-omics continues to evolve, future advancements in HF drug discovery are likely to be driven by improvements in HF phenotyping, machine learning methods, and data integration.

First, the phenotyping of HF in omics studies should be grounded in robust clinical knowledge and incorporate deep, comprehensive phenotyping. To ensure both the reproducibility and clinical relevance of findings, it is essential to standardize and harmonize HF phenotyping across studies.

Second, advanced computational tools and artificial intelligence (AI) and machine learning (ML) algorithms will play a critical role in identifying patterns across vast omics datasets, integrating demographic information with genomic, transcriptomic, proteomic, metabolomic, and epigenomic data. By uncovering hidden relationships between molecular profiles and clinical outcomes, ML models can help identify potential biomarkers and therapeutic targets, thus improving our ability to predict therapeutic responses based on a patient’s unique molecular signature and moving us toward more personalized approaches to treatment. One promising application of AI/ML approaches to multi-omics data is identifying patient heterogeneity, moving beyond the traditional approach of focusing solely on the most prevalent HF phenotypes or omics-based biomarker in a heterogeneous HF population. AI/ML methods can help uncover distinct molecular phenotypes, facilitating the tailoring of therapeutics to specific patient subclusters. In addition, AI/ML approaches can help predict the toxicity profiles of new compounds and aid in the design of more efficient clinical trials by identifying optimal dosing regimens, patient cohorts, and surrogate endpoints, thus accelerating the time to market for new therapies.

Finally, to increase the likelihood of identifying targets with high potential, it is essential to integrate multiple layers of omics data. While genomic studies alone can highlight genetic risk factors, the combination of genomics with transcriptomics, proteomics, metabolomics, and epigenomics, will provide a more holistic understanding of the molecular mechanisms driving HF. Multi-omics approaches can pinpoint the key genes, proteins, and metabolic pathways that are most likely to be involved in the disease process, improving the probability that a target is both causal and druggable. By combining these omics layers, we can create a more comprehensive map of the biological networks underlying HF, which can accelerate the identification of novel, actionable therapeutic targets.

## Figures and Tables

**Table 1:** Advantages and limitations of omics technologies in HF drug discovery.

Omics Type	Advantages	Limitations
Genomics	▪ Provides insight into the genetic architecture of HF, identifying mutations, variations, and genetic pathways relevant to HF ▪ Advances in high-throughput sequencing technologies have made genomic analyses faster and cheaper, allowing for studies with larger sample sizes ▪ Can identify individuals with a specific genetic makeup that may respond better to certain HF treatments	▪ Many genes have unknown functions, and variants may exhibit complex, multi-factorial, and pleiotropic effects ▪ Most of the human genome is composed of non-protein-coding regions, which are incompletely understood ▪ There is a long trajectory between detecting genetic associations and identifying mechanistic insights
Proteomics	▪ Can identify and monitor disease biomarkers by analyzing the proteins in urine or serum, which can aid in early diagnosis or monitoring treatment responses ▪ Can identify post-translational modifications that can contribute to HF ▪ Can provide a comprehensive map of protein interactions associated with HF pathways	▪ There can be a masking effect, where there is a reduced detection of low-abundant proteins due to the presence of high-abundant proteins ▪ Proteins can undergo many modifications, and a single gene can encode multiple protein isoforms ▪ Variability in sample preparation and technical artifacts can affect the reliability of data
Transcriptomics	▪ Measures gene expression levels, which can give insights into cellular responses, disease progression, and treatment effects ▪ Can reveal how genes are actively expressed under different HF therapies ▪ Can identify dysregulated genes in HF that may pave the way for new therapies	▪ Changes in gene expression may not always reflect functional changes ▪ There can be noise in transcriptomic data, which can lead to errors in gene expression measurement ▪ Incomplete understanding of transcriptional processes, as many studies are conducted at a single time point
Metabolomics	▪ Provides insight into the substrates and products of metabolism that drive essential cellular functions ▪ Metabolites can be correlated with HF to identify useful biomarkers and provide insight into disease mechanism ▪ Can be used in tracking how a drug is metabolized, understanding its pharmacokinetics and pharmacodynamics, and uncovering toxicity and adverse events	▪ Difficult to evaluate the biological role of an identified metabolite and determine how metabolic pathways are perturbed by a disease ▪ Variations in sample preparation, instrumentation, and analytical platform exist, challenging reproducibility efforts ▪ Current analytical methods lack the sensitivity and specificity to identify and quantify the full scope of existing metabolites
Epigenomics	▪ Offers the ability to harness the endogenous mechanisms by which cells regulate gene expression for identifying new therapeutic avenues ▪ Can reveal new epigenetic changes involved in HF progression, which may lead to new therapies ▪ Epigenetic modifications in HF can be influenced by lifestyle factors and chronic conditions, which can help to identify areas for intervention	▪ Difficult to pinpoint specific, reproducible epigenetic alterations that directly contribute to HF ▪ Challenging to develop a drug that can target a specific epigenetic modification without causing unintended consequences ▪ Difficult to establish causality between specific epigenetic changes and HF, complicating the ability to translate findings into actionable insights

## Data Availability

No new data were created or analyzed in this study.

## References

[1] Savarese G., Becher P.M., Lund L.H., Seferovic P., Rosano G.M.C., Coats A.J.S. (2022). Global burden of heart failure: A comprehensive and updated review of epidemiology. Cardiovasc. Res..

[2] Sayed A., Abramov D., Fonarow G.C., Mamas M.A., Kobo O., Butler J., Fudim M. (2024). Reversals in the Decline of Heart Failure Mortality in the US, 1999 to 2021. JAMA Cardiol..

[3] Heidenreich P.A., Bozkurt B., Aguilar D., Allen L.A., Byun J.J., Colvin M.M., Deswal A., Drazner M.H., Dunlay S.M., Evers L.R. (2022). 2022 AHA/ACC/HFSA Guideline for the Management of Heart Failure: A Report of the American College of Cardiology/American Heart Association Joint Committee on Clinical Practice Guidelines. Circulation.

[4] Carnicelli A.P., Clare R., Hofmann P., Chiswell K., DeVore A.D., Vemulapalli S., Felker G.M., Sarocco P., Mentz R.J. (2021). Characteristics and Outcomes of Patients with Heart Failure with Reduced Ejection Fraction After a Recent Worsening Heart Failure Event. J. Am. Heart Assoc..

[5] McDonagh T.A., Metra M., Adamo M., Gardner R.S., Baumbach A., Böhm M., Burri H., Butler J., Čelutkienė J., Chioncel O. (2023). 2023 Focused Update of the 2021 ESC Guidelines for the diagnosis and treatment of acute and chronic heart failure. Eur. Heart J..

[6] Ferrari R., Böhm M., Cleland J.G.F., Paulus W.J.S., Pieske B., Rapezzi C., Tavazzi L. (2015). Heart failure with preserved ejection fraction: Uncertainties and dilemmas. Eur. J. Heart Fail..

[7] Raufaste-Cazavieille V., Santiago R., Droit A. (2022). Multi-omics analysis: Paving the path toward achieving precision medicine in cancer treatment and immuno-oncology. Front. Mol. Biosci..

[8] Crowther L.M., Poms M., Plecko B. (2018). Multiomics tools for the diagnosis and treatment of rare neurological disease. J. Inherit. Metab. Dis..

[9] Ward R.A., Aghaeepour N., Bhattacharyya R.P., Clish C.B., Gaudillière B., Hacohen N., Mansour M.K., Mudd P.A., Pasupneti S., Presti R.M. (2021). Harnessing the potential of multiomics studies for precision medicine in infectious disease. Open Forum Infect. Dis..

[10] DiMasi J.A., Grabowski H.G., Hansen R.W. (2016). Innovation in the pharmaceutical industry: New estimates of R&D costs. J. Health Econ..

[11] Sun D., Gao W., Hu H., Zhou S. (2022). Why 90% of clinical drug development fails and how to improve it?. Acta Pharm. Sin. B.

[12] Rasooly D., Peloso G.M., Pereira A.C., Dashti H., Giambartolomei C., Wheeler E., Aung N., Ferolito B.R., Pietzner M., Farber-Eger E.H. (2023). Genome-wide association analysis and Mendelian randomization proteomics identify drug targets for heart failure. Nat. Commun..

[13] Minikel E.V., Painter J.L., Dong C.C., Nelson M.R. (2024). Refining the impact of genetic evidence on clinical success. Nature.

[14] Ochoa D., Karim M., Ghoussaini M., Hulcoop D.G., McDonagh E.M., Dunham I. (2022). Human genetics evidence supports two-thirds of the 2021 FDA-approved drugs. Nat. Rev. Drug Discov..

[15] Levin M.G., Tsao N.L., Singhal P., Liu C., Vy H.M.T., Paranjpe I., Backman J.D., Bellomo T.R., Bone W.P., Biddinger K.J. (2022). Genome-wide association and multi-trait analyses characterize the common genetic architecture of heart failure. Nat. Commun..

[16] Shah S., Henry A., Roselli C., Lin H., Sveinbjörnsson G., Fatemifar G., Hedman Å.K., Wilk J.B., Morley M.P., Chaffin M.D. (2020). Genome-wide association and Mendelian randomisation analysis provide insights into the pathogenesis of heart failure. Nat. Commun..

[17] Gaziano J.M., Concato J., Brophy M., Fiore L., Pyarajan S., Breeling J., Whitbourne S., Deen J., Shannon C., Humphries D. (2016). Million Veteran Program: A mega-biobank to study genetic influences on health and disease. J. Clin. Epidemiol..

[18] Joseph J., Liu C., Hui Q., Aragam K., Wang Z., Charest B., Huffman J.E., Keaton J.M., Edwards T.L., Demissie S. (2022). Genetic architecture of heart failure with preserved versus reduced ejection fraction. Nat. Commun..

[19] Rasooly D., Giambartolomei C., Peloso G.M., Dashti H., Ferolito B.R., Golden D., Horimoto A.R.V.R., Pietzner M., Farber-Eger E.H., Wells Q.S. (2025). Large-scale multi-omics identifies drug targets for heart failure with reduced and preserved ejection fraction. Nat. Cardiovasc. Res..

[20] Bozkurt B., Coats A.J., Tsutsui H., Abdelhamid M., Adamopoulos S., Albert N., Anker S.D., Atherton J., Böhm M., Butler J. (2021). Universal Definition and Classification of Heart Failure: A Report of the Heart Failure Society of America, Heart Failure Association of the European Society of Cardiology, Japanese Heart Failure Society and Writing Committee of the Universal Definition of Heart Failure. J. Card. Fail..

[21] Rasooly D., Patel C.J. (2019). Conducting a Reproducible Mendelian Randomization Analysis Using the R Analytic Statistical Environment. Curr. Protoc. Hum. Genet..

[22] Rasooly D., Peloso G.M. (2021). Two-Sample Multivariable Mendelian Randomization Analysis Using R. Curr. Protoc..

[23] Zuber V., Grinberg N.F., Gill D., Manipur I., Slob E.A.W., Patel A., Wallace C., Burgess S. (2022). Combining evidence from Mendelian randomization and colocalization: Review and comparison of approaches. Am. J. Hum. Genet..

[24] Rasooly D., Peloso G.M., Giambartolomei C. (2022). Bayesian Genetic Colocalization Test of Two Traits Using coloc. Curr. Protoc..

[25] Beauverger P., Ozoux M.-L., Bégis G., Glénat V., Briand V., Philippo M.-C., Daveu C., Tavares G., Roy S., Corbier A. (2020). Reversion of cardiac dysfunction by a novel orally available calcium/calmodulin-dependent protein kinase II inhibitor, RA306, in a genetic model of dilated cardiomyopathy. Cardiovasc. Res..

[26] Alexander V.J., Xia S., Hurh E., Hughes S.G., O’Dea L., Geary R.S., Witztum J.L., Tsimikas S. (2019). N-acetyl galactosamine-conjugated antisense drug to *APOC3* mRNA, triglycerides and atherogenic lipoprotein levels. Eur. Heart J..

[27] Ritchie S.C. (2025). Discovery of drug targets for heart failure with preserved and reduced ejection fraction. Nat. Cardiovasc. Res..

[28] Tsimikas S., Moriarty P.M., Stroes E.S. (2021). Emerging RNA therapeutics to lower blood levels of LP(a): JACC focus seminar 2/4. J. Am. Coll. Cardiol..

[29] Connelly C.M., Moon M.H., Schneekloth J.S. (2016). The Emerging Role of RNA as a Therapeutic Target for Small Molecules. Cell Chem. Biol..

[30] Franco-Serrano L., Hernández S., Calvo A., Severi M.A., Ferragut G., Pérez-Pons J., Piñol J., Pich Ò., Mozo-Villarias Á., Amela I. (2018). MultitaskProtDB-II: An update of a database of multitasking/moonlighting proteins. Nucleic Acids Res..

[31] Aebersold R., Mann M. (2016). Mass-spectrometric exploration of proteome structure and function. Nature.

[32] Cao T.H., Jones D.J.L., Voors A.A., Quinn P.A., Sandhu J.K., Chan D.C.S., Parry H.M., Mohan M., Mordi I.R., Sama I.E. (2020). Plasma proteomic approach in patients with heart failure: Insights into pathogenesis of disease progression and potential novel treatment targets. Eur. J. Heart Fail..

[33] Voors A.A., Anker S.D., Cleland J.G., Dickstein K., Filippatos G., van der Harst P., Hillege H.L., Lang C.C., Ter Maaten J.M., Ng L. (2016). A systems BIOlogy Study to TAilored Treatment in Chronic Heart Failure: Rationale, design, and baseline characteristics of BIOSTAT-CHF. Eur. J. Heart Fail..

[34] Adamy C., Mulder P., Khouzami L., Andrieu-abadie N., Defer N., Candiani G., Pavoine C., Caramelle P., Souktani R., Le Corvoisier P. (2007). Neutral sphingomyelinase inhibition participates to the benefits of N-acetylcysteine treatment in post-myocardial infarction failing heart rats. J. Mol. Cell Cardiol..

[35] Smith J.G., Gerszten R.E. (2017). Emerging Affinity-Based Proteomic Technologies for Large-Scale Plasma Profiling in Cardiovascular Disease. Circulation.

[36] McDermott J.E., Wang J., Mitchell H., Webb-Robertson B.-J., Hafen R., Ramey J., Rodland K.D. (2013). Challenges in Biomarker Discovery: Combining Expert Insights with Statistical Analysis of Complex Omics Data. Expert. Opin. Med. Diagn..

[37] Egerstedt A., Berntsson J., Smith M.L., Gidlöf O., Nilsson R., Benson M., Wells Q.S., Celik S., Lejonberg C., Farrell L. (2019). Profiling of the plasma proteome across different stages of human heart failure. Nat. Commun..

[38] Nayor M., Short M.I., Rasheed H., Lin H., Jonasson C., Yang Q., Hveem K., Felix J.F., Morrison A.C., Wild P.S. (2020). Aptamer-Based Proteomic Platform Identifies Novel Protein Predictors of Incident Heart Failure and Echocardiographic Traits. Circ. Heart Fail..

[39] Dieden A., Girerd N., Ottosson F., Molvin J., Pareek M., Melander O., Bachus E., Råstam L., Lindblad U., Daka B. (2024). Proteomic biomarkers and pathway analysis for progression to heart failure in three epidemiological representative cohorts. Eur. J. Heart Fail..

[40] Qin H., Tromp J., Ter Maaten J.M., Voordes G.H.D., van Essen B.J., André de la Rambelje M., van der Hoef C.C.S., Santema B.T., Lam C.S.P., Voors A.A. (2024). Clinical and Proteomic Risk Profiles of New-Onset Heart Failure in Men and Women. JACC Heart Fail..

[41] Nath M., Romaine S.P.R., Koekemoer A., Hamby S., Webb T.R., Nelson C.P., Castellanos-Uribe M., Papakonstantinou M., Anker S.D., Lang C.C. (2022). Whole blood transcriptomic profiling identifies molecular pathways related to cardiovascular mortality in heart failure. Eur. J. Heart Fail..

[42] Jovanovich A., Ix J.H., Gottdiener J., McFann K., Katz R., Kestenbaum B., de Boer I.H., Sarnak M., Shlipak M.G., Mukamal K.J. (2013). Fibroblast growth factor 23, left ventricular mass, and left ventricular hypertrophy in community-dwelling older adults. Atherosclerosis.

[43] Ix J.H., Katz R., Kestenbaum B.R., de Boer I.H., Chonchol M., Mukamal K.J., Rifkin D., Siscovick D.S., Sarnak M.J., Shlipak M.G. (2012). Fibroblast growth factor-23 and death, heart failure, and cardiovascular events in community-living individuals: CHS (Cardiovascular Health Study). J. Am. Coll. Cardiol..

[44] Sharma S., Joseph J., Chonchol M., Kaufman J.S., Cheung A.K., Rafeq Z., Smits G., Kendrick J., HOST Investigators (2013). Higher fibroblast growth factor-23 concentrations associate with left ventricular systolic dysfunction in dialysis patients. Clin. Nephrol..

[45] Wagner A., Cohen N., Kelder T., Amit U., Liebman E., Steinberg D.M., Radonjic M., Ruppin E. (2015). Drugs that reverse disease transcriptomic signatures are more effective in a mouse model of dyslipidemia. Mol. Syst. Biol..

[46] Ahmad T., Kelly J.P., McGarrah R.W., Hellkamp A.S., Fiuzat M., Testani J.M., Wang T.S., Verma A., Samsky M.D., Donahue M.P. (2016). Prognostic Implications of Long-Chain Acylcarnitines in Heart Failure and Reversibility with Mechanical Circulatory Support. J. Am. Coll. Cardiol..

[47] Perry A.S., Amancherla K., Huang X., Lance M.L., Farber-Eger E., Gajjar P., Amrute J., Stolze L., Zhao S., Sheng Q. (2024). Clinical-transcriptional prioritization of the circulating proteome in human heart failure. Cell Rep. Med..

[48] Paul S.M., Mytelka D.S., Dunwiddie C.T., Persinger C.C., Munos B.H., Lindborg S.R., Schacht A.L. (2010). How to improve R&D productivity: The pharmaceutical industry’s grand challenge. Nat. Rev. Drug Discov..

[49] Nguyen N., Jennen D., Kleinjans J. (2022). Omics technologies to understand drug toxicity mechanisms. Drug Discov. Today.

[50] Schirle M., Bantscheff M., Kuster B. (2012). Mass spectrometry-based proteomics in preclinical drug discovery. Chem. Biol..

[51] Selvaraj S., Fu Z., Jones P., Kwee L.C., Windsor S.L., Ilkayeva O., Newgard C.B., Margulies K.B., Husain M., Inzucchi S.E. (2022). Metabolomic Profiling of the Effects of Dapagliflozin in Heart Failure with Reduced Ejection Fraction: DEFINE-HF. Circulation.

[52] Yurista S.R., Silljé H.H.W., Oberdorf-Maass S.U., Schouten E.-M., Pavez Giani M.G., Hillebrands J.-L., van Goor H., van Veldhuisen D.J., de Boer R.A., Westenbrink B.D. (2019). Sodium-glucose co-transporter 2 inhibition with empagliflozin improves cardiac function in non-diabetic rats with left ventricular dysfunction after myocardial infarction. Eur. J. Heart Fail..

[53] Scisciola L., Taktaz F., Fontanella R.A., Pesapane A., Surina, Cataldo V., Ghosh P., Franzese M., Puocci A., Paolisso P. (2023). Targeting high glucose-induced epigenetic modifications at cardiac level: The role of SGLT2 and SGLT2 inhibitors. Cardiovasc. Diabetol..

[54] Harrington J., Fonarow G.C., Khan M.S., Hernandez A., Anker S., Böhm M., Greene S.J., Felker G.M., Vaduganathan M., Butler J. (2023). Medication-Attributable Adverse Events in Heart Failure Trials. JACC Heart Fail..

[55] Hansen J., Xiong Y., Siddiq M.M., Dhanan P., Hu B., Shewale B., Yadaw A.S., Jayaraman G., Tolentino R.E., Chen Y. (2024). Multiscale mapping of transcriptomic signatures for cardiotoxic drugs. Nat. Commun..

[56] Sharma A., Burridge P.W., McKeithan W.L., Serrano R., Shukla P., Sayed N., Churko J.M., Kitani T., Wu H., Holmström A. (2017). High-throughput screening of tyrosine kinase inhibitor cardiotoxicity with human induced pluripotent stem cells. Sci. Transl. Med..

[57] van Hasselt J.G.C., Rahman R., Hansen J., Stern A., Shim J.V., Xiong Y., Pickard A., Jayaraman G., Hu B., Mahajan M. (2020). Transcriptomic profiling of human cardiac cells predicts protein kinase inhibitor-associated cardiotoxicity. Nat. Commun..

[58] Al Sultan A., Rattray Z., Rattray NJ W. (2024). Integrative analysis of toxicometabolomics and toxicoproteomics data: New molecular insights into thiazolidinedione-induced cardiotoxicity. Metabolomics.

[59] Iorio A., Pozzi A., Senni M. (2017). Addressing the Heterogeneity of Heart Failure in Future Randomized Trials. Curr. Heart Fail. Rep..

[60] Zielinski J.M., Luke J.J., Guglietta S., Krieg C. (2021). High Throughput Multi-Omics Approaches for Clinical Trial Evaluation and Drug Discovery. Front. Immunol..

[61] Rushton C.A., Satchithananda D.K., Jones P.W., Kadam U.T. (2015). Non-cardiovascular comorbidity, severity and prognosis in non-selected heart failure populations: A systematic review and meta-analysis. Int. J. Cardiol..

[62] Bozkurt B., Aguilar D., Deswal A., Dunbar S.B., Francis G.S., Horwich T., Jessup M., Kosiborod M., Pritchett A.M., Ramasubbu K. (2016). Contributory Risk and Management of Comorbidities of Hypertension, Obesity, Diabetes Mellitus, Hyperlipidemia, and Metabolic Syndrome in Chronic Heart Failure: A Scientific Statement From the American Heart Association. Circulation.

[63] Antman E.M., Loscalzo J. (2016). Precision medicine in cardiology. Nat. Rev. Cardiol..

[64] Lavalle C., Mariani M.V., Severino P., Palombi M., Trivigno S., D’Amato A., Silvetti G., Pierucci N., Di Lullo L., Chimenti C. (2024). Efficacy of modern therapies for heart failure with reduced ejection fraction in specific population subgroups: A systematic review and network meta-analysis. Cardiorenal Med..

[65] Center for Drug Evaluation & Research (2020). Treatment for Heart Failure: Endpoints for Drug Development Guidance for Industry. U.S. Food and Drug Administration. https://www.fda.gov/regulatory-information/search-fda-guidance-documents/treatment-heart-failure-endpoints-drug-development-guidance-industry.

[66] (2021). Using machine learning approaches for multi-omics data analysis: A review. Biotechnol. Adv..

[67] Piccini J.P., Abraham W.T., Dufton C., Carroll I.A., Healey J.S., van Veldhuisen D.J., Sauer W.H., Anand I.S., White M., Wilton S.B. (2019). Bucindolol for the Maintenance of Sinus Rhythm in a Genotype-Defined HF Population: The GENETIC-AF Trial. JACC Heart Fail..

[68] Ibrahim N.E., Burnett J.C., Butler J., Camacho A., Felker G.M., Fiuzat M., O’Connor C., Solomon S.D., Vaduganathan M., Zile M.R. (2020). Natriuretic Peptides as Inclusion Criteria in Clinical Trials: A JACC: Heart Failure Position Paper. JACC Heart Fail..

[69] Solomon S.D., McMurray J.J.V., Anand I.S., Ge J., Lam C.S.P., Maggioni A.P., Martinez F., Packer M., Pfeffer M.A., Pieske B. (2019). Angiotensin-Neprilysin Inhibition in Heart Failure with Preserved Ejection Fraction. N. Engl. J. Med..

[70] Anker S.D., Butler J., Filippatos G., Ferreira J.P., Bocchi E., Böhm M., Brunner-La Rocca H.-P., Choi D.-J., Chopra V., Chuquiure-Valenzuela E. (2021). Empagliflozin in Heart Failure with a Preserved Ejection Fraction. N. Engl. J. Med..

[71] Pitt B., Pfeffer M.A., Assmann S.F., Boineau R., Anand I.S., Claggett B., Clausell N., Desai A.S., Diaz R., Fleg J.L. (2014). Spironolactone for heart failure with preserved ejection fraction. N. Engl. J. Med..

[72] Ibrahim N.E., Gaggin H.K., Konstam M.A., Januzzi J.L. (2016). Established and Emerging Roles of Biomarkers in Heart Failure Clinical Trials. Circ. Heart Fail..

[73] Pabón M.A., Cunningham J.W., Claggett B.L., Packer M., Zile M., Pfeffer M.A., Lefkowitz M., Shi V., Rizkala A., McMurray J.J.V. (2022). Natriuretic peptide-based inclusion criteria in heart failure with preserved ejection fraction clinical trials: Insights from PARAGON-HF. Eur. J. Heart Fail..

[74] Yancy C.W., Jessup M., Bozkurt B., Butler J., Casey D.E., Colvin M.M., Drazner M.H., Filippatos G.S., Fonarow G.C., Givertz M.M. (2017). 2017 ACC/AHA/HFSA Focused Update of the 2013 ACCF/AHA Guideline for the Management of Heart Failure: A Report of the American College of Cardiology/American Heart Association Task Force on Clinical Practice Guidelines and the Heart Failure Society of America. Circulation.

[75] Packer M., Fowler M.B., Roecker E.B., Coats A.J.S., Katus H.A., Krum H., Mohacsi P., Rouleau J.L., Tendera M., Staiger C. (2002). Effect of carvedilol on the morbidity of patients with severe chronic heart failure: Results of the carvedilol prospective randomized cumulative survival (COPERNICUS) study. Circulation.

[76] Motiwala S.R., Szymonifka J., Belcher A., Weiner R.B., Baggish A.L., Sluss P., Gaggin H.K., Bhardwaj A., Januzzi J.L. (2013). Serial measurement of galectin-3 in patients with chronic heart failure: Results from the ProBNP Outpatient Tailored Chronic Heart Failure Therapy (PROTECT) study. Eur. J. Heart Fail..

[77] Lanfear D.E., Luzum J.A., She R., Gui H., Donahue M.P., O’Connor C.M., Adams K.F., Sanders-van Wijk S., Zeld N., Maeder M.T. (2020). Polygenic Score for β-Blocker Survival Benefit in European Ancestry Patients with Reduced Ejection Fraction Heart Failure. Circ. Heart Fail..

[78] Topkara V.K., Mann D.L. (2011). Role of microRNAs in cardiac remodeling and heart failure. Cardiovasc. Drugs Ther..

[79] Montgomery R.L., Hullinger T.G., Semus H.M., Dickinson B.A., Seto A.G., Lynch J.M., Stack C., Latimer P.A., Olson E.N., van Rooij E. (2011). Therapeutic inhibition of miR-208a improves cardiac function and survival during heart failure. Circulation.

[80] Bocchi E.A., Vilella de Moraes A.V., Esteves-Filho A., Bacal F., Auler J.O., Carmona M.J., Bellotti G., Ramires A.F. (2000). L-arginine reduces heart rate and improves hemodynamics in severe congestive heart failure. Clin. Cardiol..

[81] Hermann H.P., Pieske B., Schwarzmüller E., Keul J., Just H., Hasenfuss G. (1999). Haemodynamic effects of intracoronary pyruvate in patients with congestive heart failure: An open study. Lancet.

[82] Ouwerkerk W., Belo Pereira J.P., Maasland T., Emmens J.E., Figarska S.M., Tromp J., Koekemoer A.L., Nelson C.P., Nath M., Romaine S.P.R. (2023). Multiomics Analysis Provides Novel Pathways Related to Progression of Heart Failure. J. Am. Coll. Cardiol..

[83] Paulus W.J., Tschöpe C. (2013). A novel paradigm for heart failure with preserved ejection fraction: Comorbidities drive myocardial dysfunction and remodeling through coronary microvascular endothelial inflammation. J. Am. Coll. Cardiol..

[84] Raphael R., Purushotham D., Gastonguay C., Chesnik M.A., Kwok W.-M., Wu H.-E., Shah S.J., Mirza S.P., Strande J.L. (2016). Combining patient proteomics and in vitro cardiomyocyte phenotype testing to identify potential mediators of heart failure with preserved ejection fraction. J. Transl. Med..

[85] Yadalam A.K., Gold M.E., Patel K.J., Liu C., Razavi A.C., Jain V., Vatsa N., Gold D., Owais M., Haroun N. (2025). Proteomics-Based Soluble Urokinase Plasminogen Activator Receptor Levels Are Associated with Incident Heart Failure Risk. JACC Adv..

[86] Thummel K.E., Lin Y.S. (2014). Sources of interindividual variability. Methods Mol. Biol..

[87] Lanfear D.E., Hrobowski T.N., Peterson E.L., Wells K.E., Swadia T.V., Spertus J.A., Williams L.K. (2012). Association of β-blocker exposure with outcomes in heart failure differs between African American and white patients. Circ. Heart Fail..

[88] Cohn J.N., Archibald D.G., Ziesche S., Franciosa J.A., Harston W.E., Tristani F.E., Dunkman W.B., Jacobs W., Francis G.S., Flohr K.H. (1986). Effect of vasodilator therapy on mortality in chronic congestive heart failure. Results of a Veterans Administration Cooperative Study. N. Engl. J. Med..

[89] Hjalmarson A., Goldstein S., Fagerberg B., Wedel H., Waagstein F., Kjekshus J., Wikstrand J., El Allaf D., Vítovec J., Aldershvile J. (2000). Effects of controlled-release metoprolol on total mortality, hospitalizations, and well-being in patients with heart failure: The Metoprolol CR/XL Randomized Intervention Trial in congestive heart failure (MERIT-HF). MERIT-HF Study Group. JAMA.

[90] CIBIS II Investigators and Committees (1999). The Cardiac Insufficiency Bisoprolol Study II (CIBIS-II): A randomised trial. Lancet.

[91] Shin J., Johnson J.A. (2010). Beta-blocker pharmacogenetics in heart failure. Heart Fail. Rev..

[92] Ibrahim N.E., Januzzi J.L. (2017). Beyond Natriuretic Peptides for Diagnosis and Management of Heart Failure. Clin. Chem..

[93] Bikdeli B., Punnanithinont N., Akram Y., Lee I., Desai N.R., Ross J.S., Krumholz H.M. (2017). Two Decades of Cardiovascular Trials With Primary Surrogate Endpoints: 1990–2011. J. Am. Heart Assoc..

[94] Zannad F., Garcia A.A., Anker S.D., Armstrong P.W., Calvo G., Cleland J.G.F., Cohn J.N., Dickstein K., Domanski M.J., Ekman I. (2013). Clinical outcome endpoints in heart failure trials: A European Society of Cardiology Heart Failure Association consensus document. Eur. J. Heart Fail..

[95] Greene S.J., Mentz R.J., Fiuzat M., Butler J., Solomon S.D., Ambrosy A.P., Mehta C., Teerlink J.R., Zannad F., O’Connor C.M. (2018). Reassessing the Role of Surrogate End Points in Drug Development for Heart Failure. Circulation.

[96] Vaduganathan M., Claggett B., Packer M., McMurray J.J.V., Rouleau J.L., Zile M.R., Swedberg K., Solomon S.D. (2018). Natriuretic Peptides as Biomarkers of Treatment Response in Clinical Trials of Heart Failure. JACC Heart Fail..

[97] Gheorghiade M., Böhm M., Greene S.J., Fonarow G.C., Lewis E.F., Zannad F., Solomon S.D., Baschiera F., Botha J., Hua T.A. (2013). Effect of aliskiren on postdischarge mortality and heart failure readmissions among patients hospitalized for heart failure: The ASTRONAUT randomized trial. JAMA.

[98] Greene S.J., Fonarow G.C., Solomon S.D., Subacius H.P., Ambrosy A.P., Vaduganathan M., Maggioni A.P., Böhm M., Lewis E.F., Zannad F. (2017). Influence of atrial fibrillation on post-discharge natriuretic peptide trajectory and clinical outcomes among patients hospitalized for heart failure: Insights from the ASTRONAUT trial. Eur. J. Heart Fail..

[99] Savarese G., Uijl A., Ouwerkerk W., Tromp J., Anker S.D., Dickstein K., Hage C., Lam C.S.P., Lang C.C., Metra M. (2022). Biomarker changes as surrogate endpoints in early-phase trials in heart failure with reduced ejection fraction. ESC Heart Fail..

[100] Packer M., McMurray J.J.V., Desai A.S., Gong J., Lefkowitz M.P., Rizkala A.R., Rouleau J.L., Shi V.C., Solomon S.D., Swedberg K. (2015). Angiotensin receptor neprilysin inhibition compared with enalapril on the risk of clinical progression in surviving patients with heart failure. Circulation.

[101] Peacock W.F., De Marco T., Fonarow G.C., Diercks D., Wynne J., Apple F.S., Wu A.H.B., ADHERE Investigators (2008). Cardiac troponin and outcome in acute heart failure. N. Engl. J. Med..

[102] Felker G.M., Mentz R.J., Teerlink J.R., Voors A.A., Pang P.S., Ponikowski P., Greenberg B.H., Filippatos G., Davison B.A., Cotter G. (2015). Serial high sensitivity cardiac troponin T measurement in acute heart failure: Insights from the RELAX-AHF study. Eur. J. Heart Fail..

[103] Pang P.S., Teerlink J.R., Voors A.A., Ponikowski P., Greenberg B.H., Filippatos G., Felker G.M., Davison B.A., Cotter G., Kriger J. (2016). Use of High-Sensitivity Troponin T to Identify Patients with Acute Heart Failure at Lower Risk for Adverse Outcomes: An Exploratory Analysis From the RELAX-AHF Trial. JACC Heart Fail..

[104] Bayes-Genis A., Aimo A., Jhund P., Richards M., de Boer R.A., Arfsten H., Fabiani I., Lupón J., Anker S.D., González A. (2022). Biomarkers in heart failure clinical trials. A review from the Biomarkers Working Group of the Heart Failure Association of the European Society of Cardiology. Eur. J. Heart Fail..

